# Effectiveness of an Online Medical Spanish Course in Improving Medical Students’ Spanish Proficiency

**DOI:** 10.7759/cureus.73863

**Published:** 2024-11-17

**Authors:** James H Moak, Jessica O González, Federico Velásquez, Vivian I Martínez, Timothy L McMurry, Erin L Keller, Weichao Chen

**Affiliations:** 1 Emergency Medicine Department, University of Virginia School of Medicine, Charlottesville, USA; 2 English Department, ASA School, Quetzaltenango, GTM; 3 Spanish Department, Celas Maya Spanish School, Quetzaltenango, GTM; 4 Data Science Department, Waymo LLC, Mountain View, USA; 5 Emergency Medicine Department, Augusta Health, Fishersville, USA; 6 Education Department, American College of Surgeons, Chicago, USA

**Keywords:** community medicine and public health, education and/or curriculum development, guatemala, hispanic/latino healthcare, immigrant health, medical spanish, minority health care, undergraduate and graduate medical education

## Abstract

Introduction: A growing need exists for language-concordant healthcare for Spanish speakers in the United States. More than three-quarters of American medical schools provide Spanish language instruction, but little data exists on best practices. The purpose of this retrospective study was to examine whether an online medical Spanish course is effective at improving medical students’ Spanish proficiency.

Materials and methods: The course involved one-on-one instruction and group mini-conferences conducted by teachers based in Quetzaltenango, Guatemala. Teachers evaluated students before and after the course using a 21-point scale adapted from the Cervantes Institute’s Diplomas of Spanish as a Foreign Language (DELE) scale. We used descriptive statistics and Wilcoxon’s signed rank test and conducted a thematic analysis.

Results: Eighty students participated. The mean (range) and median levels on the pre-course assessment were 3.8 (1-13) and 3, respectively; the post-course levels were 8.0 (3-16) and 7 (p < 0.001). Thirty-five students (43.8%) completed course evaluations. Twenty-five (71.4%) rated the course as excellent, eight (22.9%) as very good, and two (5.7%) as good.

Conclusion: Language instruction in medical Spanish conducted online by native speakers abroad is effective and well-received by medical students. Future studies should examine whether students maintain their gains in proficiency over the long term.

## Introduction

Worldwide, the number and proportion of international migrants have increased significantly over the past three decades [1]. According to the U.S. Census Bureau, Latinos make up nearly one-fifth of the U.S. population [2], and by the year 2035, this proportion may be closer to one-fourth [3]. Given recent estimates that approximately 28% of Latinos have limited English proficiency [4], language barriers between healthcare providers and Spanish speakers will persist for the foreseeable future and may place patients at greater risk for adverse outcomes [5]. A growing need exists in the U.S. for healthcare providers who are able to provide language-concordant care for their Spanish-speaking patients.

The proportion of American medical schools offering Spanish language instruction has risen in recent years [6-9]. However, the structure of these programs has been disparate, and no clear consensus exists on best practices [7,10]. Some have recommended online instruction, but no data exists to suggest this approach is useful [11]. The purpose of this study was to determine if a novel online medical Spanish course is an effective means of instruction for medical students. The results of this study were previously presented as an abstract at the annual meeting of the European Society for Emergency Medicine (EuSEM) in Berlin, Germany, on October 18, 2022.

## Materials and methods

This study was a retrospective investigation of third- and fourth-year medical students’ performance in a two-week, online medical Spanish course offered in late spring, 2020, during the early stages of the COVID-19 pandemic. The study involved medical students enrolled at the University of Virginia School of Medicine. Data were derived from pre-enrollment questionnaires designed by the U.S.-based course director, pre- and post-course language assessments conducted by the Spanish instructors in Guatemala, and course evaluations solicited by our School of Medicine. We did not conduct a sample size analysis as the maximum number of potential participants in the study was equal to our enrollment, which was capped at 80 students (40 for the first two weeks of the course and 40 for the second). The course was offered only to third- and fourth-year medical students who were furloughed from patient care activities due to the pandemic. No students were excluded from the study based on their pre-course level of fluency.

The study was approved by our Institutional Review Board (IRB) for Social and Behavioral Sciences and conducted with informed consent. The course consisted of 35 hours of one-on-one online instruction with teachers based at a Spanish school in Quetzaltenango, Guatemala (3.5 hours per day), 15 hours of online group learning through mini-conferences (1.5 hours per day), and 30 hours of homework (3 hours per day). The mini-conferences addressed topics such as Guatemalan history, culture, public health, and education.

Prior to enrollment, students categorized their level of Spanish proficiency and rated their cultural familiarity with Latin America. Students’ initial Spanish level was assessed by their teachers using a 21-point scale in use at the Spanish school since 2010. This scale is a more detailed adaptation of one used by the Cervantes Institute for the Diplomas of Spanish as a Foreign Language (DELE) exam [12]. The DELE scale is comprised of six levels: A1 (lowest), A2, B1, B2, C1, and C2 (highest). The scale used at the Spanish school expands upon the DELE scale with subcategories (e.g., A2.1, A2.2, A2.3, etc.) to yield a modified 21-point scale from A1.1 (lowest) to C2.4 (highest).

Upon course conclusion, students were asked to complete an electronic evaluation form, and their Spanish proficiency was reassessed by their Spanish instructors. For statistical analysis, students’ scores were converted to a numerical value from 1 to 21 and analyzed using statistical software (R version 4.0, R Foundation for Statistical Computing, Vienna, Austria). Preliminary histograms of these scores revealed them to be non-normally distributed. Hence, we used Wilcoxon’s signed rank test and descriptive statistics where appropriate and reported students’ mean, range, and median scores on the modified DELE. In addition, we undertook a thematic analysis of qualitative data.

## Results

All eighty students enrolled participated in the study (Table 1).

**Table 1:** Demographic characteristics of students in an on-line medical Spanish course (N = 80) ^a^Designations for pre-course Spanish language ability were based on the following definitions: early beginner = limited comprehension and vocabulary ability; late beginner = good reading and listening comprehension, basic (simple sentence) speaking ability; intermediate = can hold a conversation, some grammatical mistakes and vocabulary lapses; advanced = able to comprehend and communicate complex ideas, rarely fumble for words; fluent/native = native speaker or functionally native, no grammatical mistakes, extensive vocabulary.

Characteristic	No. (%)
Gender	
Male	42 (52.5)
Female	38 (47.5)
Level of training	
Third-year medical student	29 (36.3)
Fourth-year medical student	51 (63.8)
Pre-course Spanish language ability (self-reported)^a^	
None	6 (7.5)
Early beginner	23 (28.7)
Late beginner	23(28.7)
Intermediate	20 (25.0)
Advanced	8 (10.0)
Fluent/Native	0 (0)
“I consider myself well-acquainted with Latin American culture and its role within the U.S. and our health care system.”	
Strongly disagree	7 (8.8)
Somewhat disagree	21 (26.2)
Neutral	16 (20.0)
Somewhat agree	32 (40.0)
Strongly agree	4 (5.0)

**Figure 1:**
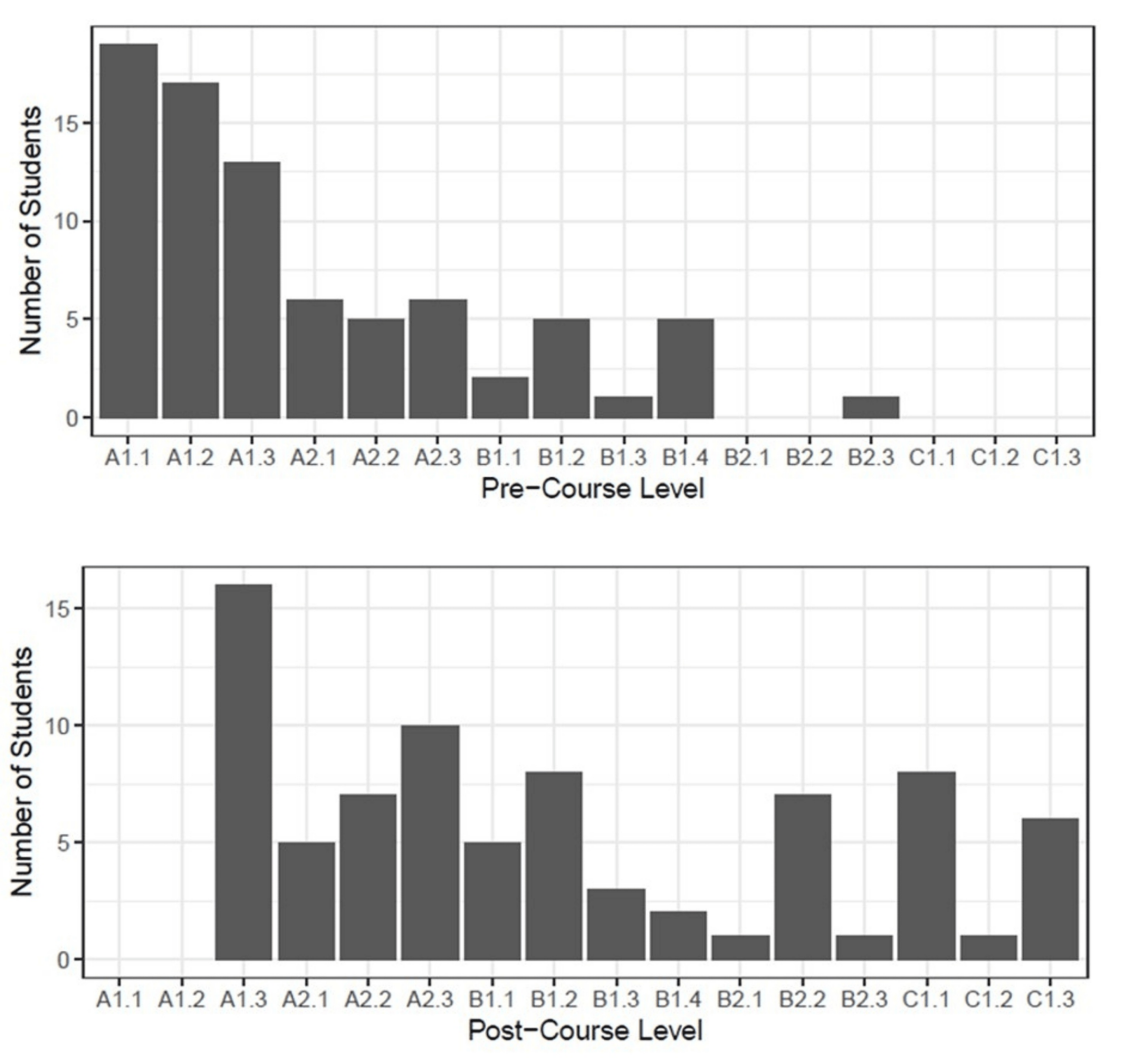
Students’ pre- and post-course Spanish language proficiency levels on the adapted Diplomas of Spanish as a Foreign Language (DELE) scale from A1.1 (lowest level = 1) to C2.4 (highest level = 21) Levels above C1.3 (level = 16) not shown as no student surpassed this level.

Pre- and post-course levels on the adapted DELE scale are illustrated in Figure 1.

**Figure 2:**
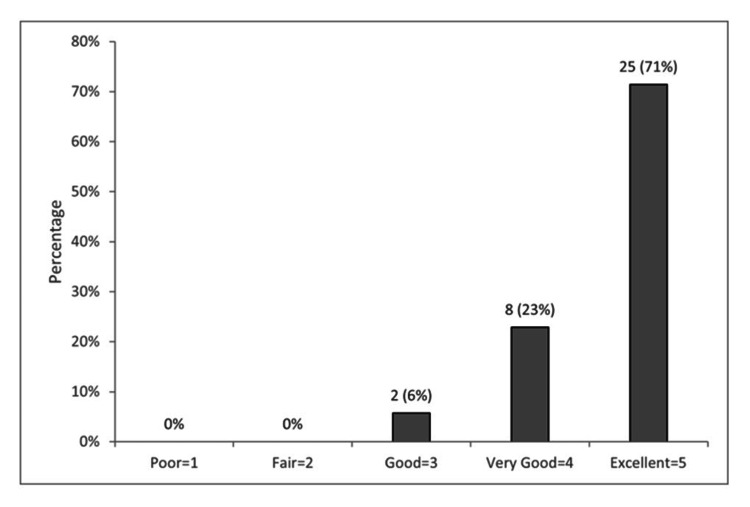
Students’ overall ratings of the online medical Spanish course (N = 35) (“Please rate the overall quality of this course")

The pre-course mean (range) and median levels were 3.8 (1-13) and 3, respectively. The post-course values were 8.0 (3-16) and 7, respectively. Wilcoxon’s signed rank test showed a statistically significant improvement in students’ modified DELE scores (p < 0.001). Thirty-five students (43.8%) completed course evaluations. On average they rated the course as a 4.66 (range 3-5) on a Likert scale from 1 (poor) to 5 (excellent) (Figure 2).

Additional evaluation data are provided in Table 2 and thematic analysis in Table 3.

**Table 2:** Student evaluation data for the online medical Spanish course (N = 35) All data presented as number (%).

Question	Yes	No	Not sure
Learning objectives clearly defined?	24 (68.6)	5 (14.3)	6 (17.1)
Assigned resources useful?	31 (88.6)	1 (2.9)	3 (8.6)
One-on-one sessions useful?	35 (100.0)	0 (0)	0 (0)
Daily mini-conferences useful?	22 (62.9)	8 (22.9)	5 (14.3)
Homework exercises helpful?	34 (97.1)	1 (2.9)	0 (0)

**Table 3:** Findings from thematic analysis of learner comments (N = 19) *Five of the seven learners who provided constructive feedback on immersive and intensive learning suggested reducing mini-conferences. These comments were also counted under the theme of mini-conferences. Two of the seven learners provided constructive feedback about the one-on-one sessions. These comments were counted under the theme of mini-conferences as well.

Themes	Representative comments	N
One-on-one sessions with the tutor	My instructor was focused on me and my goals and started by asking me what I hoped to get out of the course. He reviewed everything that was rusty for me, provided exercises related to specific vocabulary I asked to learn, and gave me timely feedback on all homework and in-class exercises. He was punctual, polite, and respectful of my time. He was so patient with me and was able to relay important concepts easily. I also just greatly enjoyed chatting with him each day and learning about life in Guatemala and his lived experiences. The tutors were accommodating and flexible with goals for this course.	Total: 15 Positive: 15 Negative: 2
Mixed opinions about the mini-conference	The mini-conferences were challenging because I did not understand a lot of what was being said, but I did find them interesting and helpful. It would be helpful to have PowerPoint presentations for all of the lectures or maybe even to send out materials regarding the lectures beforehand to help with our learning. I am at a high intermediate level but it was difficult for me to understand all of the lectures. The conferences helped particularly with training Spanish listening skills. However, for a beginner the level of Spanish was much too difficult to understand only allowing partial understanding of occasional verbs or nouns with the assistance of staff defining higher level vocabulary in the "chat" section of Zoom. If there was a creation of a more "beginner-level" conference, I believe it would be beneficial for more beginner students for comprehension practice and not just listening practice. I enjoyed most of the mini-conferences, learning about Guatemalan medicine and culture, although the one about literature was very dense.	Total: 14 Positive: 6 Constructive feedback: 14
Mixed opinions about intensiveness and immersiveness	Being able to be fully in "Spanish mode" helped us be more comfortable with our speaking. It was great to have the opportunity to speak and hear so much Spanish for two weeks. Regarding conferences, I think it would be nice if they were cut down to an hour. With the 3.5-4.5 hours of live instruction in the mornings, having a 1.5 hour afternoon session is kind of a lot of camera time. I wish the sessions with the teachers were shorter because it was overwhelming to learn so much at one time. Also I would have liked to have that time to practice via homework. Also I think the conferences could be an hour long and encourage more questions in the chat	Total: 9 Positive: 4 Constructive feedback: 7*

## Discussion

Access to language-concordant healthcare is an important social determinant of health for patients with limited language proficiency in their adopted country [5,13]. Recent mass migrations from Venezuela, Syria, Ukraine, Sudan, and other countries underscore the need for health professionals in host countries to adopt creative strategies for the provision of language-concordant care to international migrants. Hiring interpreters represents one approach but is limited by cost, training requirements, and potential for underutilization [14]. Another is to recruit and train more bilingual health professionals. This approach has intrinsic value for the promotion of cultural competency [14] and may lead to better patient outcomes [15,16]. Many investigators in the U.S. have called for expanded instruction in medical Spanish for healthcare workers [9,10,17-19]. To our knowledge, this report describes the first use of online Spanish instruction for U.S.-based medical students by native speakers abroad.

While a growing number of American medical schools provide Spanish language instruction, the approach taken varies widely [8-10]. Strategies include student-run programs, self-study courses, clerkship experiences, study abroad programs, and online instruction [8,9]. Little consensus exists on best practices [10]. Reuland recommended the following six guiding principles: 1) longitudinal program with multiple learning modalities, 2) targeted towards intermediate to advanced level students, 3) academic credit provided, 4) integrated with existing curriculum, 5) main focus on language skills with secondary emphasis on cultural issues, and 6) proficiency measured [17].

Ortega et al. later maintained that virtual strategies hold promise [11]. Our course, conducted online during the early stages of the pandemic, is the first to describe the enlistment of native speakers abroad to teach U.S.-based medical students. Consistent with expert opinion [17,20], we measured students’ pre- and post-course performance. To date, no proficiency scale for Spanish has been universally adopted by medical schools, though some have called for a national certification exam [20]. Our study is the first to incorporate a scale based on a recognized international standard, the DELE.

Of note, we disagree with Reuland’s emphasis on targeting students with intermediate to advanced proficiency [17]. More than a third of our students had zero to minimal Spanish beforehand, yet all showed improvement. We contend that medical schools should encourage appropriately tiered Spanish language training for all motivated students irrespective of prior exposure. Whether medical schools should require Spanish language instruction is subject to further debate.

Limitations

Our study is not without limitations. First, while the DELE scale is a recognized standard in Spanish language assessment, our study relied upon a modified version of the scale that may not be familiar to other instructors. Further, to our knowledge, the DELE has not been validated for health care providers per se and may not represent the ideal assessment tool for a health care setting. Audio recording of actual patient encounters may provide a more realistic appraisal of a student’s language skills in a clinical context [21]. In addition, both pre- and post-course assessments were performed by the same teacher assigned to work with a particular student for the duration of the course. Hence, teachers may have subconsciously overestimated a student’s progress on the post-course assessment. Such a phenomenon could have exaggerated the success of our course. Future studies should assess post-course proficiency in a blinded fashion. Additionally, we did not undertake a cost analysis. The negotiated costs for Spanish instruction in this course may have been unique to market forces during the pandemic. Also of interest was the rather low response rate, 43.8%, to the optional course evaluation solicited by our School of Medicine. Conceivably, students who found the course to be unhelpful neglected to complete final course evaluations. Last, this study provides no insight into long-term proficiency. Further study is needed to determine whether students in an intensive course such as ours improve or at least maintain their proficiency later in their careers.

## Conclusions

The challenge of providing language-concordant care for immigrant populations is a global one that will require a multipronged approach. The need for language-concordant care for Spanish-speaking patients in the U.S. will only increase in the coming decades. American medical schools will require innovative strategies to meet this challenge. We present an effective approach to instruction in medical Spanish involving virtual teaching for medical students by native speakers abroad utilizing a standardized scale for pre- and post-course assessments. Future studies should seek to determine whether students in an intensive course such as ours improve or at least maintain their Spanish skills over the long term.
